# Cutaneous Leishmaniasis in Tigray, Ethiopia: Clinical patterns, environmental drivers and public health implications

**DOI:** 10.1371/journal.pntd.0013994

**Published:** 2026-02-18

**Authors:** Shewaye Belay Tessema, Belay Gebreyohannes Hailu, Tadyos Hagos Embaye, Berhane Fitsum Debes, Genet Kehasi Nigus, Birhan Solomon Reda, Helen P. Price, Afework Mulugeta Bezabih

**Affiliations:** 1 Parasitology Department, Faculty of Medical Laboratory Sciences, College of Health Sciences, Mekelle University, Tigray, Ethiopia; 2 School of Medicine, College of Health Sciences, Mekelle University, Mekelle, Tigray, Ethiopia; 3 Dermatology Department, Ayder Comprehensive Specialized Hospital, College of Health Sciences, Mekelle University, Tigray, Ethiopia; 4 Dignity Period Project, College of Health Sciences, Mekelle University, Tigray, Ethiopia; 5 School of Life Sciences, Keele University, Newcastle-under-Lyme, United Kingdom; 6 School of Public Health, College of Health Sciences, Mekelle University, Tigray, Ethiopia; Advanced Centre for Chronic and Rare Diseases, INDIA

## Abstract

**Background:**

Cutaneous leishmaniasis (CL) is a vector-borne skin disease caused by the bite of female sandflies infected with the *Leishmania* parasite and is widely distributed in the highlands of Tigray, Northern Ethiopia. However, despite its widespread distribution, little is known about the actual epidemiology of the disease in Tigray and Ethiopia as a whole. Therefore, this study aimed to document the epidemiology, risk factors and public health implications of CL in Tigray, Ethiopia.

**Methods:**

Between March and May 2022, a cross-sectional household survey was conducted in seven districts of Tigray, Northern Ethiopia, using multistage sampling technique. Participants were clinically examined for scars and/or active lesions and samples taken from active lesions were investigated for parasite amastigotes by microscopy. Data on socio-demographic characteristics and environmental determinants were documented using semi-structured questionnaires. Bivariate and multivariate logistic regressions were used to identify risk factors associated with CL infections.

**Results:**

A total of 3,817 individuals (48.2% males and 51.8% females) residing in 927 households were screened and included in the study. A total of 484 individuals showed clinical evidence of CL infection (13.7% of all males, 11.5% of all females). The overall prevalence was found to be 12.7% (4.5% active lesions, 8.2% scars). Active cases were predominantly localized cutaneous leishmaniasis (LCL) (85%) but a substantial number of mucocutaneous (MCL) cases (11%) and diffused (DCL) (4%) types were also identified. The highest rates of active lesions (5.2%) were found in children aged 1–14 years. Most lesions (69%) occurred on the face, and 51.4% of cases had ≥ 2 lesions. Of the study sites screened, Emba Alaje district had the highest prevalence (15.7%). Cases clustered in highland zones (70.2% at 2,300–3,200 m altitude; 93% at 2,000–3,000 m). Significant host risk factors included age, outdoor sleeping and poor housing (cracked walls). Moreover, proximity to hyraxes, bats, caves, or animal burrows were identified among the significant environmental risk factors (p < 0.05).

**Conclusions:**

Cutaneous leishmaniasis is an underprioritized yet serious public health challenge in Tigray, driven by environmental and behavioral factors. Several forms of the disease (LCL, MCL and DCL) are prevalent in the region, with children and young adults being the most affected. Advocacy for regional recognition of CL, integration of CL control into existing NTD programmes is recommended. Targeted interventions for high-risk groups, enhanced surveillance and mapping, vector and reservoir control measures, community awareness and healthcare capacity and a One Health approach are highly recommended—for sustainable control of CL in Tigray, Ethiopia.

## Authors’ summary

Cutaneous leishmaniasis (CL) is one of the most overlooked of the neglected tropical diseases which is widely distributed in the highlands of Tigray, Northern Ethiopia. However, despite its widespread distribution, little is known about the actual epidemiology of the disease in the region. In this study, we examined the epidemiology, risk factors and public health implications of CL in Tigray. Our findings indicate that CL represents a serious public health burden in the region, with an overall prevalence of 12.7% (4.5% active lesions, 8.2% scars) and all three forms of the disease localized (LCL), mucocutaneous (MCL) and diffuse (DCL) are very prevalent. Notably, most lesions in Tigray occurred on the face, this predominance of facial lesions in Northern Ethiopia, contrasts with other CL hotspots globally. More than half of CL cases had two or more lesions per individual; but, the reason why most lesions on the face and why many cases in Ethiopia acquired ≥2 lesions needs further investigation. In the present study, all CL cases were exclusively found at altitudes between 1,997-3,164 meters above sea level. Significant host risk factors included age, outdoor sleeping and poor housing (cracked walls) and environmental risk factors such as proximity to hyraxes, bats, caves, or animal burrows within a 300-m radius were significantly associated—suggesting both outdoor and indoor transmission. Targeted interventions for high-risk groups, vector and reservoir control measures and community awareness are crucial measures needed—for sustainable control of CL in Tigray and other endemic regions in Ethiopia, towards delivering targets set out in the WHO Roadmap for NTDs (2021–2030).

## Introduction

Cutaneous leishmaniasis (CL) is a vector-borne disease caused by obligate intracellular protozoan parasites of the genus *Leishmania* and transmitted through the bite of infected female sandflies. CL is manifested in three major clinical forms: localised cutaneous leishmaniasis (LCL), characterized by single or multiple lesions; mucocutaneous leishmaniasis (MCL), affecting the mucosa of the nose and mouth; and diffuse cutaneous leishmaniasis (DCL), characterized by numerous non‐ulcerating papular, nodular, or plaque lesions [1,2]. Unlike many other CL-endemic countries, the MCL and DCL forms of the disease are believed to be relatively common in Ethiopia [1] (although poorly documented) and this causes significant clinical challenges [2]. The DCL form resembles lepromatous leprosy [1] which further complicates diagnosis and treatment of the disease.

CL is endemic in more than 90 countries, and between 0.7 to 1.2 million new cases are estimated to occur annually. Moreover, 350 million people worldwide are estimated to be at risk of infection [3,4]. Global CL incidence is increasing due to a range of factors including climatic/environmental changes [5], poverty [6], lack of treatment access and drug resistance [7], and population unrest and displacement due to war and conflicts [8–10]. However, partly due to profound underreporting among endemic countries [6], this disease remains highly neglected. In Ethiopia, CL is one of the most common neglected tropical diseases (NTD) [11], most often found in 10–15 year old children [12,13] and predominantly prevalent at altitudes ranging between 1,600 and 2,700 meters [14]. Bush hyraxes (*Heterohyrax brucei*) and rock hyraxes (*Procavia capensis)* are the known reservoir hosts of CL in the highland areas of the country [15,16].

In Ethiopia, more than 170 districts are suspected to be CL-endemic [17], 20,000–50,000 new cases are estimated to occur per year [3], and over 29 million people are estimated to be at risk of infection [2,12]. However, in 2021, only 913 CL cases were formally reported to the World Health Organization (WHO, 2022) [18] from Ethiopia, indicating huge underreporting of the disease in the country. There are some well-described CL foci areas in Ethiopia [19–22], and the overall pooled prevalence of the disease is estimated to be 20.4% [23]. However, the disease is believed to be highly underreported and more widespread than previously described [11]. The true burden of the disease is unknown due to fragmented reporting, with geographical and logistical barriers and a lack of awareness. CL is not a notifiable disease in Ethiopia, many cases of LCL do not receive formal healthcare and data from rural health posts rarely reaches national surveillance systems.

In the Tigray region of Northern Ethiopia, the prevalence of CL was investigated in the late 1930s and reported from the Adigrat highlandsas “Oriental sore in Agamé (Abyssinia)” [24]. However, relative to the other parts of Ethiopia, community-based epidemiological studies on CL in Tigray have been scarce. One study published in 2015 showed that the overall prevalence was 14.0% (6.7% for active lesions and 7.3% for scar) in Saesie Tsaeda-emba district in Eastern Tigray [19], while a prevalence of 2.3% was found for active lesions and 20.9% for CL scars in Degua-Tembien and Ganta-afeshum districts in Southeastern and Eastern Tigray in 2019 [13]. Both of these field-based studies were restricted to areas previously identified to be endemic for CL. Many sites have not been studied and the actual epidemiology of the disease and its geographic distribution, including in new potential areas, have not yet been properly established. Specifically, the epidemiology of the potentially severe MCL and DCL types of the disease, is less known across Tigray.

In order to devise and implement efficient CL intervention and control programmes, addressing evidence gaps in CL prevalence and distribution is timely and necessary from a public health perspective are crucial inputs for sound intervention and control programs. Therefore, this study was aimed to investigate the geographic distribution of the disease burden (especially MCL and DCL types) in Tigray, including in previously non-endemic areas (new catchments); and to determine major socio-demographic determinants and potential environmental factors associated with CL infections. The findings from this study may support health policymakers to devise and implement better prevention and control programs of CL in Tigray and across Ethiopia, this aligns with the WHO 2021–20230 NTDs eradication roadmap.

## Methods

### Ethics statement

Ethical clearance was obtained from the Research Ethics Committee and Institutional Review Board (IRB) of the College of Health Sciences of Mekelle University (Ref: ERC 1396/2019 and renewed on 15^th^ November, 2021; MU-IRB 2098/2021; using the same approval Ref. ERC1396/2019) and a permit letter (Ref: 882/1418/11) was granted by the Tigray Health Bureau (THB). Permissions to conduct the study in all of the study localities were obtained from each district health office. Written informed consent was obtained from all adult participants and a parent or guardian of participating minors, and a verbal assent from minor participants prior to specimen collection and delivery of questionnaires. Individuals presenting with suspected severe active LCL lesions including highly suspected cases for active LCL but found negative using slide smear microscopy and all active MCL and DCL cases were referred to Ayder Comprehensive Specialized Hospital for formal diagnosis and treatment. Except those who were referred to Ayder hospital for further diagnosis and treatment, names of participants were not recorded and there was no link between the participants and the collected data.

### Study area

The Tigray region is located in the northern part of Ethiopia between 12^O^ 15‘N and 14^O^ 57‘N latitude and 36^O^ 27‘E and 39^O^ 59’E longitude [25]. The region is administratively divided into seven (7) zones, namely Central, Eastern, Mekelle, Northwestern, Southern, Southeastern and Western zones. The region is further divided into 94 districts (locally called Woredas). The 94 woredas are further divided into 813 sub-districts (locally called Tabias). Tabias are the smallest administrative units in the region, having clearly defined boundaries and an estimated population. While the majority of the land in Tigray lies between 1,000 and 3,900 meters above sea level (m.a.s.l), the altitude varies from about 200 meters in Erob district of eastern zone to over 3,900 meters in Tsibet Mountain in the Southern zone of Tigray. The landscape of the region consists of five elevation categories: hot lowland (<500 meters), warm lowland (500–1,500 m), mid-highland (1,501–2,300 m), highland (2,301–3,200 m) and Alpine highlands (>3,200 m) [26]. The temperature of the region varies with altitude from hot to very cool and the annual average temperature is estimated to be around 20°C. The climate in Tigray also varies with altitude and the majority of the land is semi-arid zone. The region has five traditional agro-climatic zone categories: hot climate (<1,500 m), warm climate (1,500–2,000 m), tepid climate (2,000–2,500 m), cool climate (2,500–3,000 m) and very cool climate (>3,000 m a.s.l.) [25]. The current study was carried out in seven districts from three zones (Eastern, Southern, and Southeastern) of Tigray, Ethiopia.

### Study design

A cross-sectional household survey was carried out between March and May 2022 in seven districts (locally called Woredas) located in three zones of Tigray Regional State, Northern Ethiopia. The study population were residents living in seven districts, namely Degua Temben, Emba Alaje, Enderta, Ganta Afeshum, Gulomekeda, Hawzen, and Saesie Tsaeda-Emba.

### Sample size determination

Due to the scarcity of field-based epidemiological studies and limited access to treatment facilities, the actual prevalence of CL in Tigray is unknown. To estimate the maximum sample size, we assumed 50% over all (active lesion plus scars) disease prevalence. The sample size was calculated using a single population proportion formula, assuming 95% CI with 0.02 margin of error and a 1.5 design effect. The minimum sample was found to be 3,602.


$$\text{N }=\;[{\text{Z}}_{\alpha /\text{2}}^{\text{2}}\text{ P}(\text{1}-\text{P})*\text{DE}]/{\text{e}}^{\text{2}}\;=\;{\left(\text{1}.\text{96}\right)}^{\text{2}}*\text{0}.\text{5}(\text{1}-\text{0}.\text{5})\text{1}.\text{5}/{\left(\text{0}.\text{02}\right)}^{\text{2}}\;=\text{3602}$$

After adding 10% of non-response rate, the total sample size was calculated to be 3,962.

### Sampling procedure

**Fig 1:**
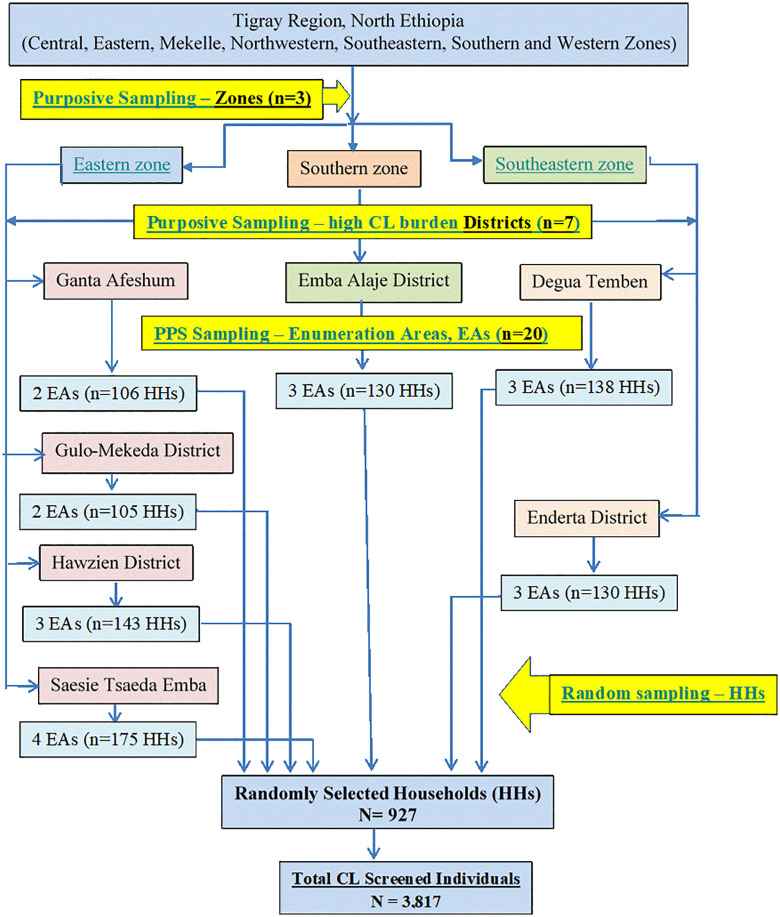
Schematic presentation of the sampling procedure. PPS, probability proportionate to size. EAs, Enumeration Areas. HHs, households.

Multi-stage cluster sampling was implemented in this study, using Tabias (sub-districts or Enumeration Areas, EAs) as clusters. Three out of the seven zones of Tigray with the highest predicted burden of CL infection were purposively selected. Using five years CL patients’ treatment data, registered at the dermatology department of Ayder Comprehensive Specialized Hospital, all catchment districts for CL-case within the selected three zones were listed. Then, seven CL catchment districts with high number of treated cases in the past five years, prior to this data collection, were selected purposively. In the next stage, utilizing data from both Central Statistics Agency [27] and Regional Bureau of Finance and Economic Development (TRBoFED) [26], cluster Tabias or Enumeration Areas (EAs) were selected using probability proportionate to size (PPS). Larger population size Tabias (EAs) were included so as to reach enough number of households (HHs) required to achieve the overall sample size needed. Finally, simple random sampling was used to select households and all household members aged six months and above were included in the present study (Fig 1).

### Data collection tools and procedures

Data was collected using clinical and parasitological diagnosis as well as a pre-tested structured questionnaire. A community member who had been previously infected with CL and left with visible scar was part of the survey team in each cluster Tabia; serving as a gatekeeper during house-to-house data collection.

### Clinical case definitions

Skin surfaces and mucous membrane of oral and nasal cavities of study participants were carefully examined for scar(s) or active lesion(s). CL is well identified by the community using its local name known as *‘Gizwa’* in the local Tigrigna language. The term *‘Gizwa’* was very helpful during clinical diagnosis in all of the study districts. Visible marks of healed lesions resulting from past infections were referred to as ‘CL scars’ using a case definition of areas of mottled and depressed skin, pigment change (either hypo-or hyper-pigmented) and smooth or shiny skin surface. Cases of active LCL were clinically defined by the presence of crater-like skin lesions with a raised border or local edema, crusty or ulcerative lesions with or without satellites, as previously described [28]. Lesions involving the mucosal regions, often with erythema and metastasizing ulcerations of the face (nose, mouth, and throat), including the nasal septum were defined as MCL lesions. Active lesions with multiple non-ulcerative, popular, nodular, or plaque were clinically defined as DCL type cases [2,29].

Infected individuals were examined for characteristic skin lesions as described above (active, healed scar or both) and the anatomic location and number of lesions were recorded. Participants with active lesions were evaluated for the type of lesion (LCL, MCL and/or DCL) and were asked about the estimated time they had had the lesion in days/months.

When any family member in a household was identified as having an active lesion or CL scar, the individual was given a code number, recorded as living in a ‘ case-household’ and the location coordinates and altitude of the house were recorded. Heads of each household or eligible adults in case-households were invited to complete a structured questionnaire, delivered by the researcher and piloted on 25 respondents. The questionnaire included questions on patient socio-demographics, e.g., age, gender, education level, occupation; and behavioral and environmental variables such as working and sleeping practices (e.g., staying in the field until late evening or overnight, outdoor sleeping), livestock ownership, whether hyrax and bats were found close to the house. The research team also physically observed each case-household and documented the housing status (e.g., presence of wall cracks/holes) and environmental features, including proximity to caves, gorges, and animal burrows.

### Parasitological diagnosis

Skin snip specimens were collected from a subsample of identified active LCL lesion cases, all active MCL suspected cases, and all active DCL type suspected cases. These were transported to the lab, smeared onto microscope slides and stained using 10% Giemsa before microscopic examination for the presence/absence of *Leishmania* amastigotes [29]. Participants who presented with the signs of active MCL, DCL and with relatively severe LCL pathology, including those who found negative for parasite amastigotes using slide smear microscopy, were referred to Ayder Comprehensive Specialized Hospital and linked to the dermatology unit of the hospital for further diagnosis and treatment. The formal diagnosis outcomes of the referred cases were collected from the dermatology unit of the hospital, with consent.

### Data analysis

The collected datasets were inputted into EPI Info version 20 and analyzed using SPSS version 23 (SPSS, IBM Inc., Chicago). Of the referred cases, those with a negative diagnosis for leishmaniasis at Ayder Comprehensive Specialized Hospital were excluded from the data analysis. Descriptive statistics were expressed as percentages and as a mean and standard deviation (SD) for categorical and continuous variables, respectively. Logistic regression was used to test the association between risk factors and the dependent variable. A univariate analysis was performed with all the variables included in the study, and those with p < 0.25 were included in the multivariate analysis. Odds ratio was calculated with 95% confidence interval for each variable and inferential statistics was set at the level of significance, p < 0.05.

## Results

### Sociodemographic characteristics of study participants

In this study, a total of 3,817 individuals (48.3% males and 51.7% females) from 927 households were approached for visual screening for CL (presence of active lesions and scars), with a 96% response rate. The minimum, maximum and median age of the participants were 2, 80 and 28 years (IQR = 12 – 35 years), respectively. About 43% of the participants were between 1–14 years of age and 27.5% were 15–29 years of age. More than 30% had not received any form of education, while about 60.4% of the respondents had received some formal education. From those who had formal education (n = 2,307), about 34% had attended primary school (1–6 grades), about 25% had received secondary level education (7–10 grades) and about 2% (n = 69) had completed college or university education. About 38% of the respondents (n = 1,443) were farmers in occupation, engaged in subsistence agricultural practices. About 58.4% of the participants were preschool or schoolchildren. A majority of the participants (66.1%) acknowledged that they had outdoor sleeping habits (Table 1).

**Table 1:** Socio-demographic and economic characteristics of the respondents from the study communities in Tigray, Northern Ethiopia, 2022 (n = 3,817).

Variables	Category	Frequency	Percent
Gender	Male	1,844	48.3
	Female	1,973	51.7
Age	1-14	1,642	43.0
	15-29	1,051	27.5
	30-44	544	14.3
	≥45	580	15.2
Education	Preschooler (<7 years age)	337	8.8
	Primary school	1,294	33.9
	Secondary school	944	24.7
	Diploma & above	69	1.8
	Unable to read & write	1,173	30.7
Occupation	Farmer	1,443	37.8
	Student/child/	2,230	58.4
	Civil servant	63	1.7
	Daily laborer	66	1.7
	Merchant/Pity trade/	15	0.4

### Environmental characteristics of the study households

In the present study, of the total (n = 927) surveyed households, 68.2% (n = 632) owned livestock and about 65% (n = 602) of the resident houses had cracks/holes in their walls. Most of the houses in the study sites were built from stone with mud plastered walls, tin or grass roofs and dust floor (S3 Appendix). Over 63% of the surveyed households were situated near to caves and/or gorges (within 300 meters) and about 65% of households were close to visible animal burrow/s (within 300 meters). Furthermore, the presence of hyrax (S3 Appendix) and bats close to the house (within 300 meters) were acknowledged by about 66%. 64% and 51% of the surveyed household respondents respectively (Table 2).

**Table 2:** Environmental characteristics of the study households within the study districts in Tigray, Northern Ethiopia, 2022 (n = 927). *Near the house refers to a distance of 300 meters or less.

Variables	Category	Frequency(n)	Percent
Household owns livestock (n = 927)	Yes	632	68.2
	No	295	31.8
Outdoor sleeping habit	Yes	2,523	66.1
	No	1,294	33.9
presence of cracks/holes in walls of the house(n = 927)	Yes	602	64.9
	No	325	35.1
Cave/gorge near* the house	Yes	587	63.3
	No	340	36.7
Animal burrow near* the house	Yes	599	64.6
	No	328	35.4
Hyrax near* the house	Yes	608	65.6
	No	319	34.4
Bat near* the house	Yes	473	51.0
	No	454	49.0

### Prevalence of CL in the study sites

In the present study, of the total individuals screened (n = 3,817), 12.7% (484/3,817) of them showed visible signs of active or previous CL, with 4.5% (173) having one or more active lesions and 8.2% (311) having one or more characteristic scars. Of the suspected active lesion cases (n = 173), the prevalence of localized (LCL), mucocutaneous (MCL) and diffuse (DCL) forms were 85.0%, 11.0% and 4.0%, respectively. Furthermore, 22% (n = 38) of the active lesion cases also had one or more CL scars left from past infection; i.e., they had both active lesion and scar, suggesting that previous infections are not sufficient to induce immunity to the parasite. When the data is further disaggregated by households, about 36% (n = 330) of the 927 households contained one or more family member who was currently or had previously been affected by CL (either active lesion and/or scar): these were considered in the datasets as “case-households”. From the total identified “case-households” (n = 330), the majority (73%) had a single case per household, 15% had two cases and 13% had three or more cases(lesions and/or scars) per household, respectively (Table 3).

**Table 3:** Burden of CL cases (visible characteristic lesions and scars) in the study participants (n = 3,817) and households (n = 927) from the study districts in Tigray, Northern Ethiopia, 2022. * = Altitude measurements [minimum 1,997 meters and maximum 3,164 meters] taken from case-households.

Variables	Category	Frequency (n)	Percent
Participants with signs of CL (active lesion and/or scar)	Yes	484	12.7
	No	3,333	87.3
Participants with active lesion	Yes	173	4.5
	No	3,644	95.5
Participants with CL scar	Yes	311	8.2
	No	3,506	91.8
Participants with both lesion and scar (n = 173)	Yes	38	22.0
	No	134	78.0
Types of active CL lesion (n = 173)	LCL	147	85.0
	MCL	19	11.0
	DCL	7	4.0
Households with at least one CL case (active and/or scar) (n = 927)	Yes	330	35.6
	No	597	64.4
Frequency of active or previous cases (active lesion or CL scar) per household (n = 330)*	One	241	73.0
	Two	48	14.5
	Three	23	7.0
	Four	12	3.6
	Five	6	1.8
Frequency of case-households in different agro-climatic zones (n = 330)*	Warm (<2,000 m)	3	0.9
	Tepid (2,000–2,499 m)	150	45.5
	Cool (2,500–3,000 m)	152	46.1
	Very cool (>3,000 m)	25	7.6
Frequency of CL cases in different agro-climatic zones (n = 484)	Warm (<2,000 m)	4	0.8
	Tepid (2,000–2,499 m)	200	41.3
	Cool (2,500–3,000 m)	250	51.7
	Very cool (>3,000 m)	30	6.2
Frequency of CL cases at a range of altitude (n = 484)	Warm lowland (500–1,500 m)	0	0.0
	Mid-highland (1,501–2,300 m)	144	29.8
	Plateau highland (2,301–3,200 m)	340	70.2
	Alpine highlands (>3,200 m)	0	0.0

### Prevalence of CL (lesions and scars) in different population groups

The study found a slightly higher prevalence of CL in males (13.7%) than females (11.5%), with more active lesions (4.9% vs. 4.1%) and scars (8.9% vs. 7.4%). Among the identified active cases, 1.2% of males and 0.8% of females showed visible signs of previous CL infections. The highest prevalence of active lesions was found in children (1–14-years old) (13.3%) and young adults (15–29-year-olds) (12.9%), with pre-school (<7 years age) children having the highest overall CL infection rate (15.4%) (Table 4).

**Table 4:** Prevalence of CL infection (active lesion & scar) among the various socio-demographic characteristics of the study communities surveyed in Tigray, Northern Ethiopia, 2022 (n = 3817).

Variables & Categories	Examined Individuals		CL infection status						Total**	
			Lesion only		Scar only		Lesion + Scar			
	N*	%	n	%	N	%	N	%	n	(%)
**Gender**										
Male	1,844	48.3	91	4.8	165	8.9	23	1.2	256	13.7
Female	1,973	51.7	82	4.1	146	7.4	15	0.8	228	11.5
**Age group**										
1-14	1,642	43.0	85	5.2	134	8.2	20	1.2	219	13.3
15-29	1,051	27.5	47	4.5	89	8.5	8	0.8	136	12.9
30-44	544	14.3	17	3.1	48	8.8	4	0.7	65	11.9
≥45	580	15.2	248	4.1	40	6.9	6	1.0	64	11.0
**Educational background**										
No formal education	1,173	30.7	14	1.2	56	4.8	3	0.3	70	6.0
Preschooler (<7 years age)	337	8.8	24	7.1	28	8.3	5	1.5	52	15.4
Primary school	1,294	33.9	81	6.3	113	8.7	18	1.4	194	15.0
Secondary school	944	24.7	51	5.4	111	11.8	12	1.3	162	17.2
Diploma & above	69	1.8	3	4.3	3	4.3	0	0.0	6	8.7
**Districts surveyed**										
Degua Temben	567	14.9	27	4.8	55	9.7	5	0.9	82	14.5
Emba Alaje	536	14.0	30	5.6	54	10.1	7	1.3	84	15.7
Enderta	536	14.0	20	3.7	28	5.2	6	1.1	48	9.0
Ganta Afeshum	438	11.5	23	5.3	33	7.5	6	1.4	56	12.8
Gulomekeda	432	11.3	20	4.6	22	5.1	6	1.4	42	9.7
Hawzen	589	15.4	23	3.9	47	8.0	4	0.7	70	11.9
Saesie Tsa/emba	719	18.8	30	4.2	72	10.0	4	0.6	102	14.2

*N = number of screened individuals in each category; n = number of cases with active lesion and/or scar.

**: Percentages do exceed 100% because of double counting in the lesion + scar category.

### CL prevalence per districts and household altitude

**Fig 2:**
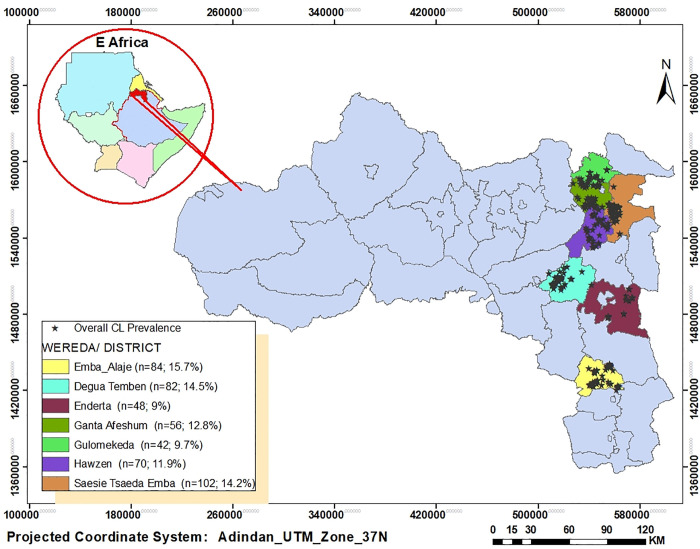
Maps showing the prevalence of CL (active lesion and scar) among study districts in Tigray, Ethiopia. The base map used is obtained from an openly available source Base maps of east African shapefile and administrative boundaries including Ethiopia were (*available at:* https://open.africa/dataset/ethiopia-shapefiles, https://open.africa/dataset/africa-shapefiles).

Significant variations were observed in CL prevalence (both active lesions and scars) across different districts and altitude ranges. Among the seven districts surveyed, Emba Alaje showed the highest overall prevalence of active CL lesions and/or scars (15.7%), followed by Degua Temben (14.5%), Tsaeda Emba (14.2%), and Ganta Afeshum (12.8%). The highest prevalence of active lesions was found in Emba Alaje (5.6%), with Ganta Afeshum (5.3%) and Degua Temben (4.8%) ranking second and third (Fig 2).

### CL distribution across the various altitude ranges

**Fig 3:**
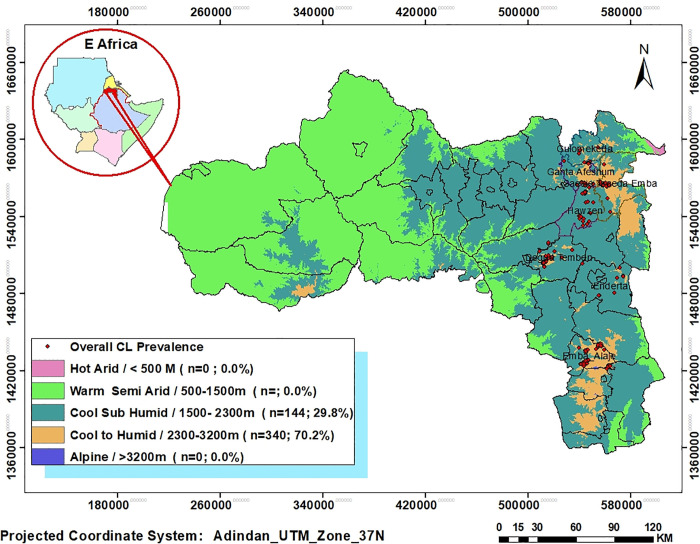
Maps showing distribution of CL cases at a range of altitudes of the study districts and agro-climatic zones in Tigray, North Ethiopia. The base map used is obtained from an openly available source *Elevation data were obtained from the ALOS PALSAR Radiometrically Terrain Corrected Digital Elevation Model with 12.5 m spatial resolution, provided by the Alaska Satellite Facility, NASA Earth data (available at:* *https://search.asf.alaska.edu). Base map of east African shapefile and administrative boundaries were obtained from open AFRICA datasets (available at:* *https://open.africa/dataset/africa-shapefiles) including Ethiopia (https://open.africa/dataset/ethiopia-shapefiles). The specific CL prevalent locations indicated in this map were developed using coordinate and elevation data of this survey and created using ArcGIS 10.8.2 software.*

All CL cases in this study were exclusively found at altitudes between 1,997-3,164 meters above sea level elevation (Partial view of the study sites’ landscape has indicated in S4 Appendix). About 30% (n = 144) and 70% (n = 340) of CL cases were within mid- highland (1,500–2,300m) and plateau highland (2,300–3,200m) elevation zones, respectively. While no CL case was found in the warm lowland (500-1500m) and Alpine highland (>3200m) elevation zones (Fig 3).

#### Type of CL active lesions identified in the study sites.

**Fig 4:**
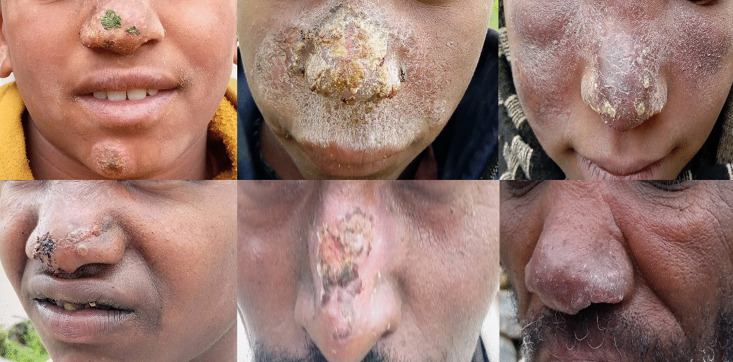
Active MCL cases in Tigray, north Ethiopia. Photographs used with participants’ consent.

Of the total active lesion cases (n = 173) detected in this survey, a majority of 85% (n = 147) were visually classified as localized cutaneous leishmaniasis (LCL) type cases. Approximately 11% (n = 19) were identified as mucocutaneous leishmaniasis (MCL) cases and 4% (n = 7) of them were diffuse cutaneous leishmaniasis (DCL) type cases. Fig 4 shows examples of MCL-type lesions identified in the study sites.

Children aged 1–14 years accounted for 49% (85/173) of all CL cases identified (including LCL, MCL, DCL types) and 53% (20/38) of the cases with both active lesions and scars. Of the active cases found, 49% presented with single lesions, while 21%, 20%, and 10% had two, three, or four or more lesions, respectively (Fig 5). The lesions primarily appeared on the face (68.8%), particularly on the nose (44.3%) and cheeks (22.4%), with 27.2% of nasal cases occurring in 1–14-year olds. The median lesion duration was 6.3 months (IQR: 3–12), with 38% of cases appearing within 6 months and 13% persisting beyond 18 months (Table 5).

**Table 5:** Active CL lesion cases in different age groups: type of lesion, number of lesions per individual, anatomic distributions, and estimated duration of the active lesion among participants (n = 173).

	Age of participants (years)								Total	
	1-14		15-29		30-44		≥45			
	n	%	N	%	n	%	n	%	N	%
**Type of active lesion**										
LCL	73	42.2	41	23.7	14	8.1	19	11.0	147	85.0
MCL	6	3.5	6	3.5	2	1.2	5	2.9	19	11.0
DCL	6	3.5	0	0.0	1	0.6	0	0.0	7	4.0
Subtotal	85	49.1	47	27.2	17	9.8	24	13.9	173	100
**Active lesion with scar left from past infection**										
Yes	20	11.6	8	5.2	4	2.3	6	3.5	38	22.0
No	65	37.6	39	22.0	13	7.5	18	10.4	135	78.0
# of active lesions per patient*										
One	41	23.7	19	11.0	14	8.1	10	5.8	84	48.6
Two	15	8.7	13	7.5	1	0.6	8	4.6	37	21.4
Three	17	9.8	11	6.4	1	0.6	6	3.5	35	20.2
Four and above	11	6.4	5	2.9	1	0.6	0	0.0	17	9.8
**Duration of lesion↔**										
< 6 months	31	17.9	13	7.5	8	4.6	13	7.5	65	37.6
6-12 months	28	16.2	19	11.0	4	2.3	7	4.0	58	33.5
12-18 months	14	8.1	10	5.8	2	1.2	2	1.2	28	16.2
>18 months	12	6.9	5	2.9	3	1.7	2	1.2	22	12.7
**Location of lesion/s**										
Face	55	31.8	32	18.5	16	9.2	16	9.2	119	68.8
Upper limbs	15	8.7	8	4.6	0	0.0	4	2.3	27	15.6
Lower limbs	8	4.6	3	1.7	1	0.6	2	1.2	14	8.1
Two or more locations	7	4	4	2.3	0	0	2	1.2	13	7.5
**Location of facial lesions**										
Cheek	20	11.6	13	7.5	5	2.9	7	4.0	45	22.4
Nose	47	27.2	20	11.6	10	5.8	12	6.9	89	44.3
Forehead	7	4.0	3	1.7	0	0.0	4	2.3	14	7.0
Ear	12	6.9	3	1.7	2	1.2	1	0.6	18	9.0
Lip	5	2.9	2	1.2	1	0.6	1	0.6	9	4.5
Chin	10	5.8	5	2.9	0	0.0	5	2.9	20	10.0
Eye	3	1.7	2	1.2	0	0.0	1	0.6	6	3.0

# = number; max = maximum; med (IQR) = median interquartile range. * = Number of active lesion per individual patient [max = 6 and med (IQR) = 2(1–3)]; ↔ = Duration of active lesion in month [max = 36, med (IQR) = 6.3 (3–12)].

**Fig 5:**
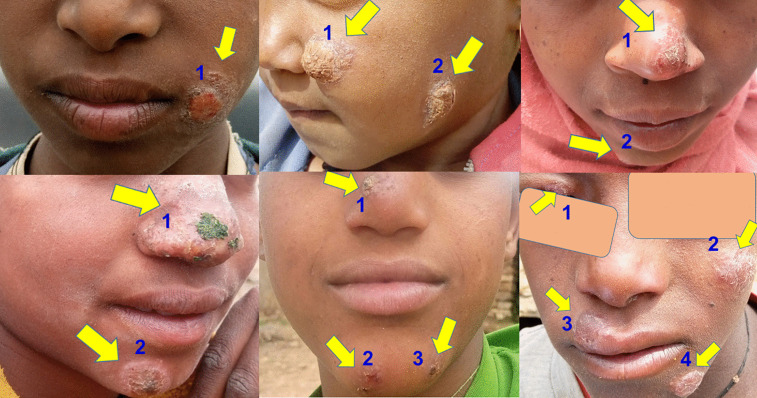
Active CL cases with one or more lesions. Photographs used with participants’ consent.

#### Geographic distribution of active LCL, MCL and DCL types.

**Fig 6:**
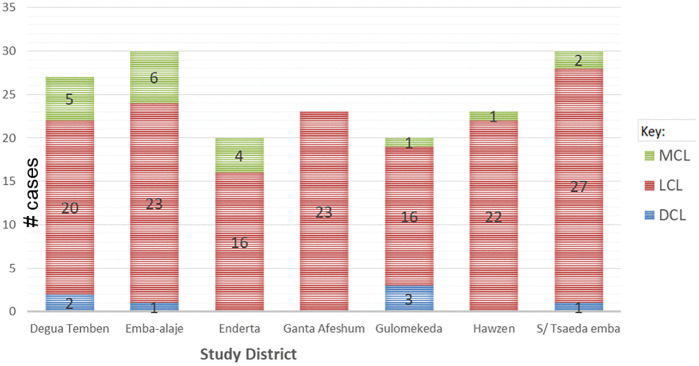
Distribution of active LCL, MCL and DCL lesions in the seven surveyed districts in Tigray, Ethiopia.

In the present study, there was a geographic variation in the prevalence of active LCL, MCL, and DCL type cases in the seven study districts. About 79% (15/19) of the active MCL cases were concentrated in three of the seven surveyed districts, namely Enderta, Emba Alaje and Degua Temben. Similarly, of the total 7 active DCL cases, 43% (3/7) of these were found in Gulomekeda and 29% (2/7) were in Degua Temben district. In contrast, all cases from Ganta Afeshum district were LCL type only (Fig 6).

### Active lesion cases prevalence per altitude ranges

**Fig 7:**
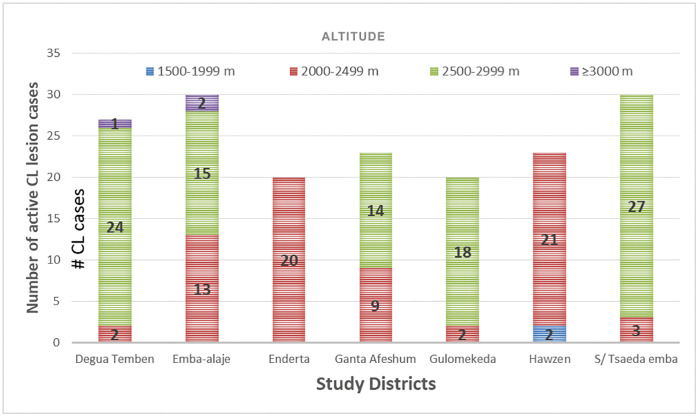
Distribution of active CL lesion across the different altitude ranges of the study districts in Tigray, Ethiopia.

The survey showed that active lesion prevalence varied by altitude across the seven districts. In Hawzen and Enderta, most cases (91.3% and 100%, respectively) were relatively at lower elevations (2,000–2,499 m). In contrast, Saesie Tsaeda Emba, Gulomekeda, and Degua Temben had most cases (88.9–90%) at higher altitudes (2,500–2,999 m). Emba Alaje and Ganta Afeshum showed wider distributions, with cases split between both altitude ranges (e.g., 50% vs. 43.3% in Emba Alaje; 60.9% vs. 39.1% in Ganta Afeshum) (Fig 7).

### Results of slide smears and referral cases

#### Results of slide smears.

68.1% (49/72) of the slide smears were positive for *Leishmania* parasite amastigotes and 31.9% (23/72) of the slide smears were negative among the specimen taken from active LCL cases (n = 72). Similarly, 70% (14/ 20) of the MCL case slide smears and 62.5% (5/8) of the DCL slide smears were positive for amastigotes among the specimens taken from active MCL cases (n = 20) and DCL cases (n = 8), respectively (Table 6).

**Table 6:** Results of slide smears using a microscope and diagnosis outcomes of referral cases at Ayder Comprehensive Specialized Hospital in Tigray, Northern Ethiopia, 2022.

Category	Examined slides	Positive		Negative	
	N	n	%	n	%
LCL	72	49	68.1	23	31.9
MCL	20	14	70	6	30.0
DCL	8	5	62.5	3	37.5
Referred CL type	#Referred cases	# Treated at the hospital		#Ruled out at the hospital	
	N	n	%	n	%
LCL	35	35	100	0	0.0
MCL	20	19	95	1*	5.0
DCL	8	7	87.5	1 Ǿ	12.5

* = Treated for psoriasis at Ayder hospital, Ǿ = Treated for leprosy at hospital

#### Results of referral cases.

Out of 35 active LCL cases with severe pathology (n = 12) and those who found negative using slide smear microscopy (n = 23) that were referred to Ayder Comprehensive Specialized Hospital, all received pentavalent antimonial treatment for CL. Among 20 MCL cases, one was diagnosed as psoriasis and excluded, leaving 19 (95%) treated. Of 8 DCL cases, 7 (87.5%) were treated, while one was confirmed as leprosy and excluded. In total, two cases (1 MCL, 1 DCL) were ruled out and excluded from the data analysis (Table 6).

### Risk factors for CL

In the present study, individuals in the age group of 1–14 years old ages were 3.6 times more likely to have CL (P < 0.001) compared to individuals in the age group of 45 years and above (Table 7). For the 15–29 years age group, this was 3.3 times more likely and for the 30–44 years age group, this was 2.3 times more likely (Table 7).

**Table 7:** Univariate and Multivariate analysis to evaluate the potential determinants of CL infection (lesion and scar) among study households (n = 927) and communities (n = 3,817) in Tigray, Northern Ethiopia, 2022.

Variable	Category	N (%)	CL infection status		Univariate analysis	Multivariate analysis	
			No (%)	Yes (%)	COR (95% CI)	AOR (95% CI)	P-value
Age group	1-14	1,642	1,430 (87.1)	212 (12.9)	0.29(0.16-0.54	3.56 (1.96-6.49)	<0.001
	15-29	1,051	907 (86.3)	144 (13.7)	0.24 (0.13-0.44)	3.32 (1.34-6.99)	<0.001
	30-44	544	475 (87.3)	69 (12.7)	0.44 (0.22-0.8	2.31 (1.16-4.60)	0.018
	≥45	580	521 (89.8)	59 (10.2)	1	1	
Gender	Male	1,844	1,593 (86.3)	256 (13.7)	1.01 (0.76-1.36)	1.00 (0.75-1.34)	0.989
	Female	1,973	1,740 (88.5)	228 (11.5)	1	1	
Outdoor sleeping	Yes	2,523	2,185 (86.6)	338 (13.4)	0.28 (0.20-0.38)	3.58 (2.61-4.90)	<0.001
	No	1,294	1,148 (88.7)	146 (11.3)	1	1	
Cracked walls in house (n = 927)	Yes	602	273 (45.3)	329 (54.7)	0.58 (0.42-0.79)	1.50 (1.11-2.02)	0.008
	No	325	170 (52.3)	155 (47.7)	1	1	
Household owns livestock	Yes	632	314 (49.7)	318 (50.3)	1.16 (0.83-1.63)	0.76 (0.55-1.05)	0.099
	No	295	129 (43.7)	166 (56.3)	1	1	
Cave/gorge/ NH	Yes	587	259 (44.1)	328 (55.9)	0.62(0.45-0.84)	1.64 (1.21-2.23)	0.002
	No	340	184 (54.1)	156 (45.9)	1	1	
Hyrax NH	Yes	608	275 (45.2)	333 (54.8)	0.70 (0.52-0.95)	1.40 (1.04-1.90)	0.028
	No	319	168 (52.7)	151 (47.3)	1	1	
Bat near House	Yes	473	198 (41.9)	275 (58.1)	0.48(0.35-0.67)	2.05(1.49-2.82)	<0.001
	No	454	245 (54.0)	209 (46.0)	1	1	
Animal burrow	Yes	599	269 (44.9)	330 (55.1)	0.42 (0.30-0.59)	2.31(1.65-3.23)	<0.001
	No	328	174 (53.0)	154 (47.0)	1	1	

Individuals who had regular outdoor sleeping habits were 3.6 times more likely to get CL infections compared to those who never had such outdoor sleeping practices. Participants who lived in substandard houses where the walls had holes/cracks were 50% more likely to have CL compared to those who resided in houses without cracked walls (Table 7).

Participants who lived in houses nearby to animal burrow/s (within 300 m) were nearly 2.3 times more likely to have CL compared to those living in residences further away from animal burrow/s. Similarly, participants who lived in residences in proximity to caves/gorges were 64% more likely to have CL compared to those living in more distant households. In addition, the presence of hyrax and bat colonies within a 300 m radius proximity to residential houses was significantly associated with CL infection (Table 7). Participants who lived close to hyrax colonies were 40% and those who lived nearby to bats residing places were 2 times more likely to get CL infection compared to those who lived in houses far away from hyraxes and bat colonies, respectively (Table 7).

## Discussion

This study was aimed to evaluate the overall epidemiology, risk factors and public health implications of CL in Tigray, Ethiopia. The study showed that the overall prevalence of CL infection (both active lesions and scars) in the study communities in Tigray was 12.7%, with 4.5% having active lesions and 8.2% showing visible scars indicative of a previous infection (S1 Appendix). Of the total active cases (n = 173) identified in the study communities, 11.0% and 4.0% of the participants with suspected CL presented with lesions that were characteristic of MCL and DCL type disease. All cases were concentrated in two climatic zones only, with 70.2% and 29.8% of CL cases found in the highland (2,300–3,200 m) and mid-highland (1,500–2,300 m) elevation zones, respectively. Risk factors for CL in the study sites included the participants’ age, outdoor sleeping, cracked walls in residential buildings, proximity of caves/gorges near to homes and the presence of hyrax, bat colonies and animal burrows close to homes.

The prevalence of active CL lesions (4.5%) found in Tigray was higher than earlier community-based studies carried out in different parts of Ethiopia. In line with the present study findings, an active lesion prevalence of 2.5% was reported from Gamo administrative zone, southwestern Ethiopia [30]; 2.05% in Sayo district, western Ethiopia [20] and 2.3% in Degua Tembien and Ganta Afeshum districts of Tigray, northern Ethiopia [13]. The present higher prevalence of active lesion could be attributed to the war in Tigray, which was erupted in 2020 [31]. On the other hand, much higher than the current findings were reported in other parts of Ethiopia. Active lesion prevalence of 18.4% was reported from Lay-Gayint, north-central Ethiopia [32] and 31.1% from Sekota, northeast Ethiopia [33]. The discrepancies could be due to differences in study settings used. Unlike to the present community-based study, both of the previous studies were hospital based studies with groups of patients as participants. Another community-based study undertaken in Saesie Tsaeda-emba district of Tigray also reported a higher (6.7%) active lesion prevalence of CL [19]. This variation could be attributed to the differences in geographic coverage of the study sites. Unlike to the present study carried out in seven endemic and non-endemic districts, the previous study was carried out in one prevously endemic district, Saesie Tsaeda-emba.

In the current study, about 22% of the participants had active lesion/s and/or healed scar/s left from past infections. In line with the present finding, a recent study report from a clinical setting in north-central Ethiopia implicated that about 39% of patients had a previous history of CL treatment [34]. In addition, a previous report from a clinical setting at the dermatology center at Ayder Comprehensive Specialized Hospital in Mekelle, Tigray reported that 2.5% of all patients receiving treatment for C in the hospital had scar/s left from past infections [35]. Moreover, a study report from Ochollo, Ethiopia, has indicated about 28% of the patients with active CL lesions had both a healed scar and an active lesion [36]. Furthermore, a recent study report from Iraq has indicated that 6.2% of the active cases had previous CL infection histories [37]. The presence of both active lesion and CL scars in individuals indicates that resolution from active lesion does not confer full immunity against reinfections with *Leishmania*. Moreover, earlier studies in North-West Ethiopia implicate that relapse of CL was common among treated cases [42], which strengthens the current finding. Furthermore, a systematic review report by van Henten S, et al., (2018) highlighted that resolution of a CL lesion would not be fully protective against re-appearance of lesions through life time [1].

CL lesions are commonly seen on body parts that are exposed and vulnerable to sandfly vector bites. In the present study, over three quarters of CL lesions were located on facial areas and 44.3% of the lesions were found on the nose. These findings are consistent with a study in the Sayo District of Western Ethiopia, where 80% of CL lesions were on the facial areas and 31.8% of lesions were specifically on the nose [20]. A recent study in Addis Ababa, Ethiopia also indicated that 89.4% of lesions were found on the facial areas and that only 5.0% of lesions were on limbs (upper and lower) and 5.6% were on multiple body regions [2]. Earlier studies reported consistent findings with the current findings, where 84·9% of active lesions in Ochollo [36] and 78·9% of lesions in Silti [38] were noticed on the facial areas.

However, unlike the prevalence of facial lesions in Ethiopia, different findings have been reported in other CL-endemic countries. A study carried out in Ardestan of Isfahan, Iran, indicated that only 5.8% of lesions were on the face, while 45.5% and 38.1% of the lesions were noticed on the hands and legs, respectively [39]. Similarly, in Hubuna of Najran, Saudi Arabia, 31.4% and 39.4% of lesions were on upper and lower extremities, respectively and only 22.6% of lesions were present on the face [40]. The reasons why an unusually high proportion of CL lesions in Ethiopia are found on the face is unknown. This could potentially be due to differences in preference of biting location by the sandfly vector or differences in environment and time of biting (e.g., bites during outdoor sleeping) or a combination of multiple factors.

This study found that among 173 active cases in the study sites in Tigray, 11% had the characteristics of MCL and 4% were DCL, showing that these severe forms of the disease are common in the region. Earlier community-based studies in Ganta Afeshum [13] and Saesie-Tsaedaemba districts of Tigray [19] reported only LCL, with no MCL or DCL cases. However, hospital-based studies in Tigray, particularly from Ayder Comprehensive Specialized Hospital (ACSH), has reported much higher MCL (25.7% - 43%) and DCL (22.9%) prevalence [35,41]. While clinical settings may overrepresent severe cases [1], the hospital data support the current findings of significant numbers of MCL and DCL cases in the region.

This study’s findings adds to a considerable body of evidence that MCL and DCL are present in Ethiopia in substantial numbers. A community-based study in Aleku Area of Sayo District, Western Ethiopia, reported that 13.6% of CL cases were MCL [20]. Besides, 34.4% MCL and 3.0% DCL cases were reported in a recent hospital-based study in ALERT hospital, Addis Ababa [2] and 4.5% of DCL cases were indicated in a previous hospital-based study in Gondar [42]. A hospital-based study report carried out among patients in Boru Meda Hospital (BMH) in Dessie indicated that 6.9% and 3.9% of all treated cases were MCL and DCL patients, respectively [21] and a similar study undertaken in Tefera Hailu Memorial Hospital (THMH) in Sekota has also indicated that 5.3% and 1.1% of all patients treated were MCL and DCL cases, respectively [33]. These findings, from regions with similar geographical settings to Tigray, support the current study’s results, indicating that both MCL and DCL are common in Ethiopia [43] and highlighted the public health significance of both disease forms in Ethiopia.

The study found that CL had the highest prevalence in younger age groups (1–14 years), followed by 15–29-year-olds, with significantly lower rates in individuals aged 45 and above. This aligns with previous studies in Ethiopia [12,13,19], and in different endemic localities in Saudi Arabia, and Iraq [37,40,44,45] which reported higher frequency of CL in children under 15. Possible reasons include increased exposure due to outdoor activities (e.g., herding, fetching water, and firewood collection) in high-risk areas like gorges and bushes—where sandflies and hyraxes (reservoir hosts) are common—especially during peak sandfly biting times in the evening. High prevalence could also be linked to immune response, with long-term adaptive immunity developing after initial infections [46]. However, our finding that some individuals have both active lesions and scars suggests that infections may not lead to complete protection. Overall, the results of the current study suggest that children under 15 are the most vulnerable group, which could be due to both behavioral exposure and immunological factors.

In the present study, all CL cases were concentrated in two of the five altitude categories of Tigray; with 70.2% and 29.8% cases found in the highland (2,300–3,200 m) and mid-highland (1,500–2,300 m) elevation zones, respectively (Table 3 and Fig 3). These findings are consistent with a study carried out in North-Central Ethiopia, where 73.5% and 26% of CL cases were found in the highland and mid-highland altitude ranges [34]. Unlike the current study, which detected CL cases between 1,997m and 3,164m, a previous study in north-central Ethiopia reported cases across a wider altitude range (1,358m–3,017m), including hot lowlands [34]. This difference may be due to expanding sandfly habitats into lowland areas, differences in vector strains or the travel history of infected individuals from endemic highlands to lowlands. This suggests there may be ecological or behavioral factors influencing CL distribution beyond just altitude.

The Tigray region has five traditional agro-climatic zones [25]. In the current study, about 93% of CL cases were found clustered within two of its five agro-climatic zones, with 51.7% occurring in the cool zone (2,500–3,000 m altitude) and 41.3% in the semi-cool/tepid zone (2,000–2,500 m). Only a small proportion of cases were detected at higher elevations above 3,000 m (6.2% of total prevalence) and in warm lowlands below 2,000 m (just 4 cases). This distribution pattern contrasts with findings from other CL-endemic countries. For example, a study in Iran reported high CL prevalence in lowland areas between 600–1,800 m [47], while research in Sri Lanka indicated a random spatial distribution of cases based on GIS analysis [48]. These geographical differences in CL distribution likely reflect variations in vector sandfly and parasite species, animal reservoir hosts, and local environmental conditions suitable for sandfly breeding and survival across different study areas.

The presence of hyrax colonies in the vicinity of residential buildings was highly associated with CL. In agreement with this finding, epidemiological studies undertaken in Dessie, Amhara region of northeast Ethiopia, has indicated that the presence of hyraxes close to houses was significantly associated with CL infections [49]. Earlier epidemiological studies carried out in Saesie Tsaeda-emba and Ganta-afeshum districts of Tigray region indicated that the presence of CL cases in households was closely associated with the presence of hyrax colonies in the vicinity [13,19]. A systematic and meta-analysis report has also indicated that the presence of hyraxes close to houses was a risk factor for CL [23].

In the present study, outdoor sleeping was significantly associated with occurrence of CL, compared to those who did not follow this practice (Table 7). In agreement with the current findings, a community-based study in Dembidolo, Oromia [50] and an epidemiological study in Tigray have also indicated that CL infection was significantly associated with outdoor sleeping habits [41]. We also found that dwelling in houses proximate to animal burrows and caves/gorges were significantly associated with CL. Similar findings were previously reported in an epidemiological study in Tigray [19]. The physical environment around houses could be serving as a favorable habitat for these reservoir hosts (including hyraxes) and insect vectors (sandflies) of the *Leishmania* parasite. Previous studies in Ochollo, SNNPR and southwestern Ethiopia have reported that transmission predominantly takes place in outdoor habitats and humans are believed to be accidental hosts in the transmission cycle [15,16]. Considering these risks, it is important that communities living in endemic regions should be made aware that nighttime outdoor activities, particularly near to caves/gorges and animal burrows may increase their exposure to *Leishmania* infections.

While outside sleeping was associated with infection, we also found that people living in houses with walls containing cracks/holes had a significantly higher risk of CL compared to those in houses with undamaged walls, suggesting peridomestic transmission. This aligns with a recent study demonstrating that sandflies (*P. longipes)* frequently feed on humans indoors, particularly in substandard housing [11]. The authors also added that in rural areas where housing status is poor, the main sites where female sandflies resided were linked with seasonality (p < 0.001). In the wet season, the majority of sandflies were captured inside buildings, while in the dry season, they resided both inside and outside houses, with the majority found outside [11]. Poor housing conditions have been linked to increased risk of CL transmission in two additional studies in Ethiopia [19,21]. Moreover, a study in southwestern Ethiopia indicated that humans were the predominant blood meal sources for sand flies indoors [15], implicating the possibility of a human-to-human transmission cycle. This evidence underscores the importance of improving housing quality to reduce exposure and interrupt potential peridomestic transmission cycles. This also challenges the traditional view that CL in Ethiopia is solely zoonotic, proposing instead that both anthroponotic and zoonotic transmission may occur. However, further research is needed to confirm whether humans are at greater risk indoors or during outdoor activities near caves. Additional evidence is also required on sandfly species, their preferred habitats, and seasonal variations in transmission to develop effective control strategies for CL in Tigray and across Ethiopia.

The current study also found that individuals living near bat colonies had a higher risk of CL, suggesting bats could play a role in disease transmission. While bats have been confirmed as natural reservoirs in South America (e.g., Brazil), evidence in Africa, including Ethiopia, remains limited but points to possible bat involvement [51–55]. Bats and sandflies often share habitats such as caves, increasing transmission opportunities, with the bat acting as an incidental host [56]. In addition the known reservoir host, the hyrax, may also share these habitats, adding to the complex transmission pattern. Further research is needed to determine the exact role of bats in the transmission dynamics of *Leishmania*.

### Strength and limitations of the study

This is the first published study to report the prevalence of MCL and DCL disease forms using field-based studies conducted among 3,817 participants living in 927 households of 7 districts located in 3 zones of Tigray, northern Ethiopia. This study also determined the altitude-based distribution of CL cases and variations of disease prevalence per elevation ranges among the studied districts in Tigray region. Given that, this study also had number of limitations. First, this was a cross-sectional survey, which limit us to infer specific causalities of the disease and different clinical forms (LCL, MCL and DCL) manifestations. Second, we have used slide smeared specimen from lesions and microscope visualization of parasite amastigots to diagnose active CL cases, without further confirmatory methods for negative result slides; hence, false-negativity among slide smeared specimen was inevitable. Therefore, the actual incidence of the disease and the magnitude its prevalence in the Tigray region of northern Ethiopia may be underestimated.

## Conclusion and recommendations from this study

The findings from this study show that CL represents a serious public health burden in Tigray, Ethiopia, with an overall prevalence of 12.7% (4.5% active lesions, 8.2% scars). Notably, 11% of active cases were found to be mucocutaneous (MCL) and 4% diffuse (DCL), highlighting that all three forms of the disease (LCL, MCL, and DCL) are prevalent in Tigray. Ethiopia is not currently listed by WHO as one of the countries with the highest prevalence of MCL but our findings and evidence from several other studies indicates that Ethiopia should be included in this list.

We found that CL cases were concentrated in highland (70.2%) and mid-highland (29.8%) zones, with key risk factors including proximity to hyrax colonies, bat habitats, animal burrows/caves, poor housing (cracked walls), and outdoor sleeping—suggesting both zoonotic (outdoor) and potential anthroponotic (indoor) transmission. The predominance of facial lesions (75%) in Tigray contrasts with other CL hotspots globally. This could be due to unique sandfly biting behaviors in Ethiopia but needs further investigation. Children (1–14 years) showed the highest CL prevalence, likely due to a combination of outdoor exposure and immature immunity. Hence, sound surveillance through improved diagnosis, active case detection, early treatment and vaccine developments are priorities. While hyraxes remain primary reservoirs, proximity to bats was found to be a risk factor, warranting further study regarding their role in disease transmission.

We have identified multiple areas that require further investigation around the ecology of transmission of *Leishmania* in Ethiopia. In addition, work is urgently needed to address the high burden that CL places on remote rural communities and to increase awareness of risk factors leading to spread of the disease. Addressing these gaps will be critical in order to design effective interventions in Tigray and other endemic regions around the globe, towards delivering targets set out in the WHO Roadmap for NTDs (2021–2030).

## Supporting information

S1 Appendix CL scars (one or more) on cases’ facial areas. Photographs used with participants’ consent.
(TIF)

S2 Appendix Status of houses from some of the study sites. (TIF)

S3 Appendix Hyraxes’ photographs collected from Study sites. (TIF)

S4 Appendix Landscape of study sites, a partial view. (TIF)
