# Supplementary figures and images for: Cutaneous Leishmaniasis in Tigray, Ethiopia: Clinical patterns, environmental drivers and public health implications
Source: PLoS Negl Trop Dis. 2026 Feb 18;20(2):e0013994. doi: 10.1371/journal.pntd.0013994 (PMC12928583; doi:10.1371/journal.pntd.0013994)

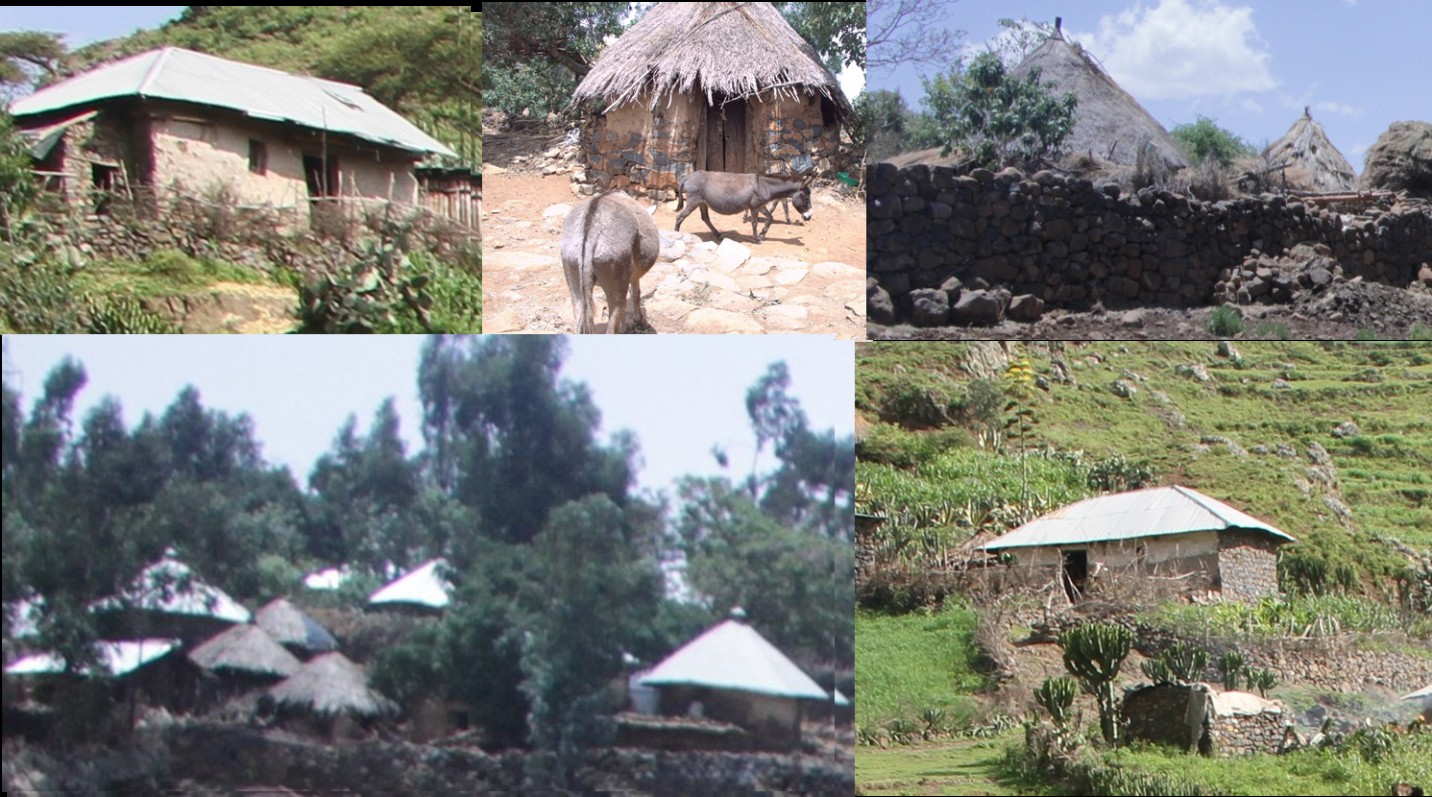

Supplement: S2 Appendix — (TIF) [file pntd.0013994.s002.tif]

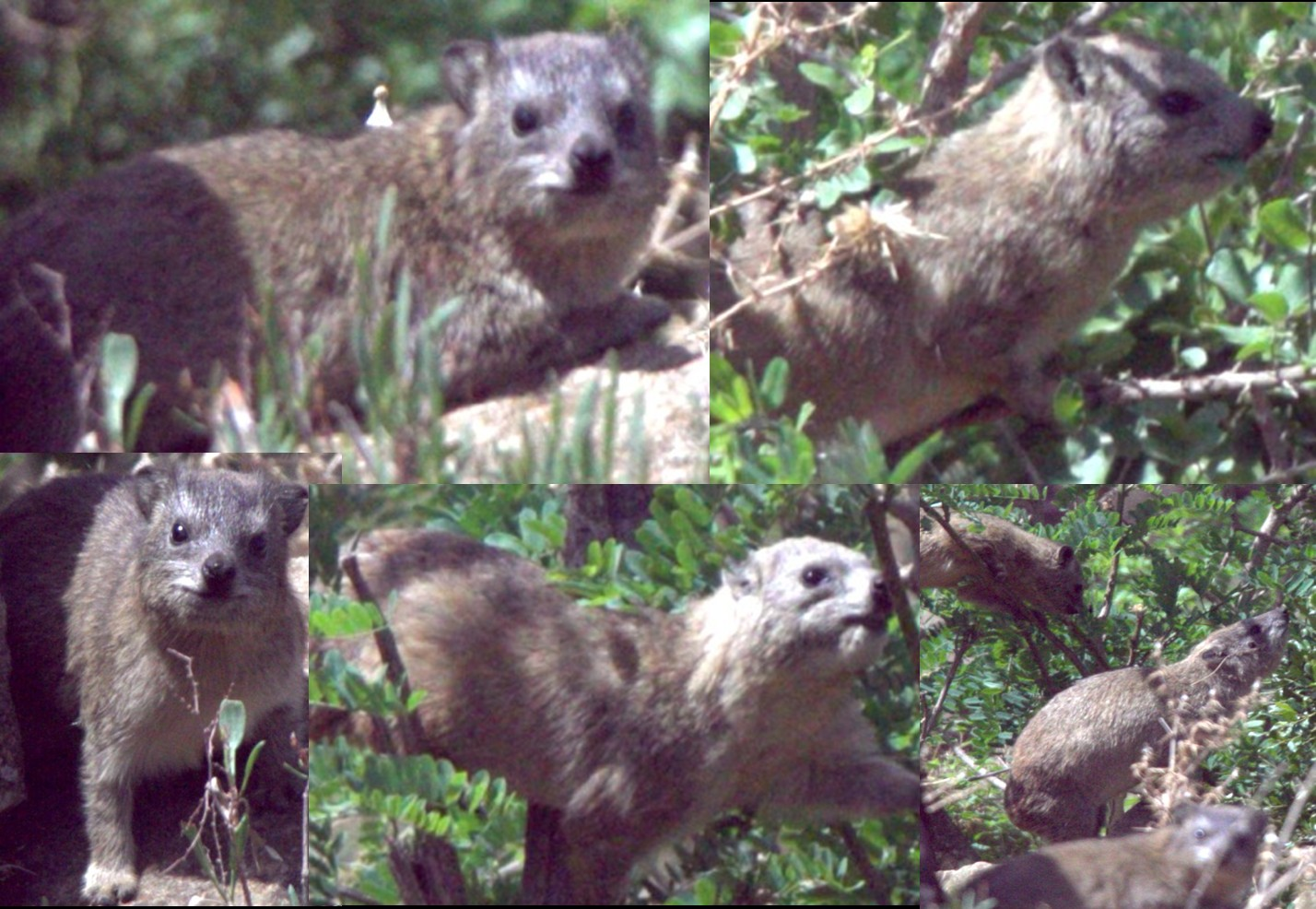

Supplement: S3 Appendix — (TIF) [file pntd.0013994.s003.tif]

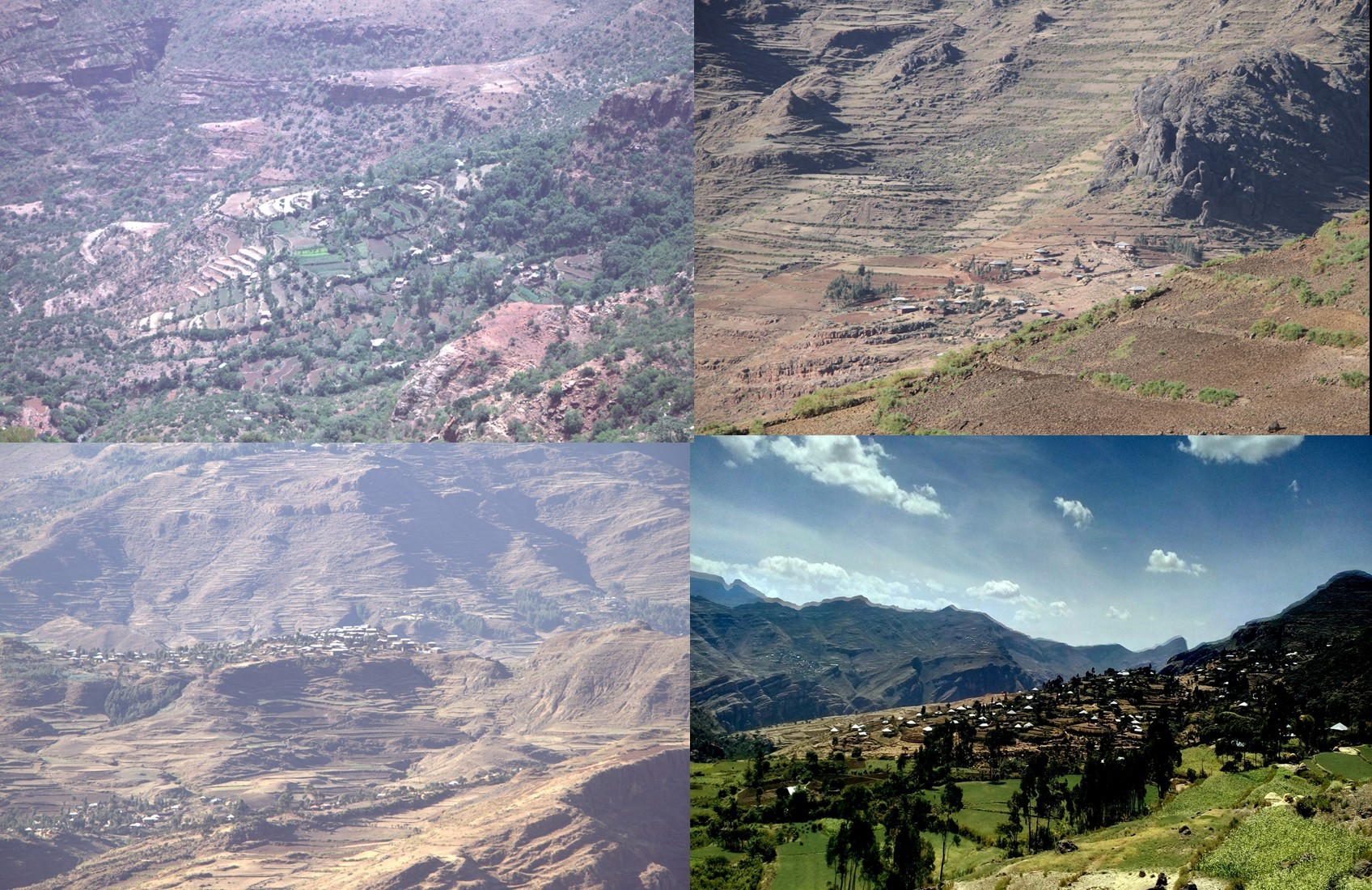

Supplement: S4 Appendix — (TIF) [file pntd.0013994.s004.tif]
